# Effects of Sex Steroids on Fish Leukocytes

**DOI:** 10.3390/biology7010009

**Published:** 2018-01-09

**Authors:** Elena Chaves-Pozo, Alfonsa García-Ayala, Isabel Cabas

**Affiliations:** 1 Centro Oceanográfico de Murcia, Instituto Español de Oceanografía, 30860 Puerto de Mazarrón, Spain; elena.chaves@ieo.es; 2 Department of Cell Biology and Histology, Regional Campus of International Excellence “Campus Mare Nostrum,” University of Murcia, 30100 Murcia, Spain; icabas@um.es

**Keywords:** oestrogens, androgens, progestins, immune system, leukocytes, teleosts

## Abstract

In vertebrates, in addition to their classically reproductive functions, steroids regulate the immune system. This action is possible mainly due to the presence of steroid receptors in the different immune cell types. Much evidence suggests that the immune system of fish is vulnerable to xenosteroids, which are ubiquitous in the aquatic environment. In vivo and in vitro assays have amply demonstrated that oestrogens interfere with both the innate and the adaptive immune system of fish by regulating the main leukocyte activities and transcriptional genes. They activate nuclear oestrogen receptors and/or G-protein coupled oestrogen receptor. Less understood is the role of androgens in the immune system, mainly due to the complexity of the transcriptional regulation of androgen receptors in fish. The aim of this manuscript is to review our present knowledge concerning the effect of sex steroid hormones and the presence of their receptors on fish leukocytes, taking into consideration that the studies performed vary as regard the fish species, doses, exposure protocols and hormones used. Moreover, we also include evidence of the probable role of progestins in the regulation of the immune system of fish.

## 1. Introduction

Steroids regulate several biological processes, including embryonic development, sex differentiation, reproduction, metabolism, circadian rhythms and immune and stress response in vertebrates [1]. Regarding the immune response, it has been known for many years that steroids act on all immune cell types through steroid receptor-dependent and receptor-independent mechanisms [2]. Recently, the presence of a great variety of substances, both natural and anthropogenic, in aquatic media has aroused interest in determining the effect of steroids on different aspects of fish biology, including the immune response. These structurally diverse substances have been shown to disrupt the function (blocking- or super-activating hormone receptors), the levels (interfering with hormone biosynthesis and metabolism) or the distribution of endogenous hormones and are, as such, classified as endocrine disrupting chemicals (EDCs) [3,4]. The EDCs, which interfere with hormone biosynthesis and metabolism, act either as inhibitors of relevant steroidogenic enzymes, or at expression levels [5]. In the International Program on Chemical Safety (IPCS) 2012, EDCs were defined as “an exogenous substance or mixture that alters function(s) of the endocrine system and consequently causes adverse health effects in an intact organism, or its progeny, or (sub) populations”; those substances disrupting the steroid regulation can be designated as xenosteroids, with further subclasses of xenoestrogens or xenoandrogens [6].

**Figure 1:**
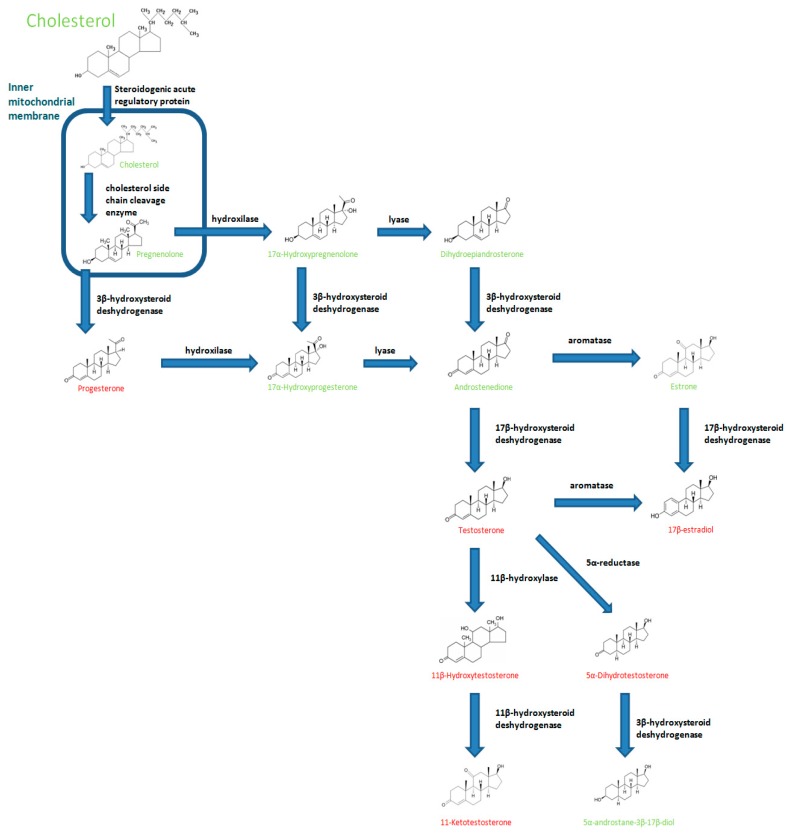
Schematic representation of the key steps involved in fish sex steroids (written in red) production (modified from [23]).

Steroidogenesis occurs primarily in different peripheral tissues like the gonads, the interrenal gland and the brain [1,7], which produce both oestrogens (C18 steroids) and androgens (C19 steroids). A schematic representation of the key steps involved in steroidogenesis in teleosts is shown in Figure 1. All classes of steroid hormones are synthesized de novo from the common precursor cholesterol [8], which is imported by the steroidogenic acute regulatory protein (StAR) into the inner mitochondrial membrane in a rate-limiting reaction [9]. Downstream of the synthesis pathway, several enzymes modify the steroid nucleus and add and modify functional groups through hydroxylation, reduction, or oxidation reviewed by [1]. Although 17β-estradiol (E_2_) is the major oestrogen in females, it is also important for normal male reproduction [10]. In fish, detectable levels of E_2_ have been reported during the reproductive cycle of male specimens of several fish species [10,11,12]. As regards androgens, dihydrotestosterone (DHT) is one of the most physiologically important androgens in many male vertebrates; in fish, it has recently been demonstrated that DHT plays a role in early development and reproduction [13]. Moreover, in fish, testosterone (T), 11-ketotestosterone (11KT) and 11β-hydroxytestosterone (OHT) are found in serum or testes of several species [14]. The third group of sex steroid hormones consists on progestins, which have traditionally been related with the final events of maturation of fish gametes [15,16]; however, it has also been demonstrated their role in the early stages of spermatogenesis [17]. Sex steroid production and modification, as well as serum steroid levels, are regulated by different parameters such as substrate availability, rapid changes in steroidogenic enzyme activity, the regulation of transcription levels of these enzymes and the amount of sex steroids binding proteins in blood and tissues [14,18]. All the biological processes including immunity are under the influence of seasonal changes such as photoperiod and temperature and reproductive conditions [19,20]. Interestingly, the seasonality associated with immunity has been related to several hormone such as melatonin, cortisol and sex steroids [19,21]. Most of the data revealed that seasonal changes in sex steroid hormones modulate several immune activities in serum and immune tissues but also revealed that peripheral immune activities such as the gonadal immune responses depends on the reproductive conditions of the specimens [12,22].

**Figure 2:**
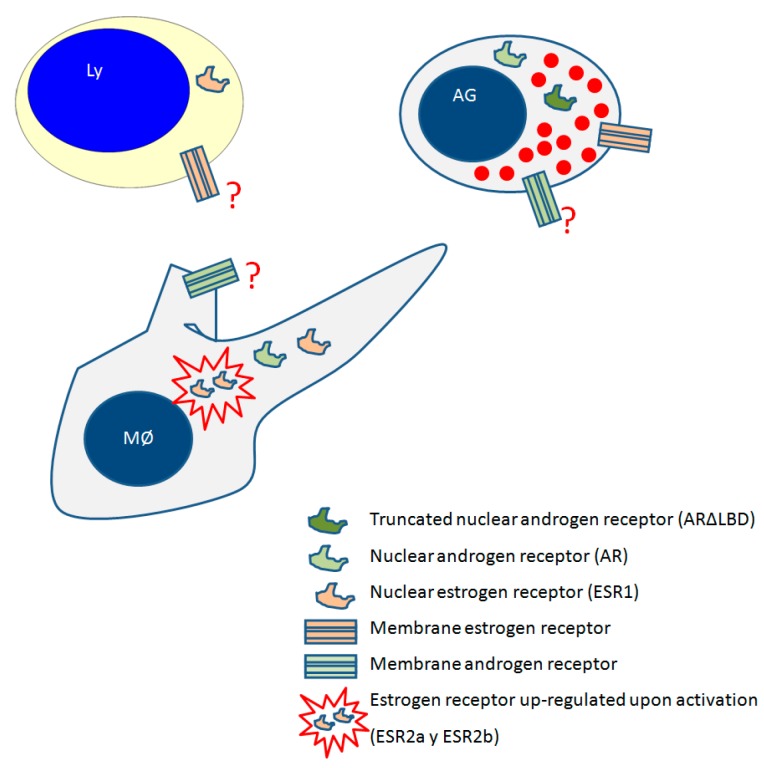
Schematic representation of the presence of oestrogen and androgen receptors in fish leukocytes accordingly to functional and gene expression data obtained in gilthead seabream. Ly, lymphocyte; AG, acidophilic granulocyte; MØ, macrophage; probable receptor location accordingly to functional data but not demonstrated to date.

The presence of specific sex steroid receptors on fish immune cells implies direct steroid-mediated immune regulation [24]. Two different types of sex steroids receptors have been described: (i) the classical nuclear receptors, which act as transcriptional regulators of genes containing oestrogen response elements in their promoter regions [25] and (ii) the membrane receptors, which are G protein–coupled receptors that mediate a rapid signalling effect of sex steroids [26]. A schematic representation of the presence of oestrogen and androgen receptors on fish leukocytes accordingly to functional and gene expression data obtained in gilthead seabream is shown in Figure 2.

As regards the immune system, teleost fish was the first evolutionary group to possess both innate and adaptive immunity response [27]. The innate immune system is considered essential for fish as they are aquatic free-living organisms from the early embryonic stages of life, poikilothermics and with a limited immunoglobulin repertoire [27,28]. Some studies have pointed out that the immune response of both wild and farmed fish can be influenced by a variety of parameters, among which temperature, stress management, fish density, light, water quality, salinity, food or immunostimulants should be mentioned [29,30,31,32,33,34].

In innate immunity, the epithelium and mucosal tissues represent the physical barriers, while phagocytes (granulocytes and monocytes/macrophages), non-specific cytotoxic cells and eosinophilic cells, including mast cells, represent the cellular effectors; meanwhile, a variety of molecules (the complement, acute phase proteins, antimicrobial polypeptides, natural antibodies and cytokines, between others) direct the humoral immune response. The main mechanisms of the innate immunity developed in teleost fish to destroy harmful stimuli include activities such as degranulation, chemotaxis, phagocytosis, production of reactive oxygen intermediates (ROIs) and nitric intermediate production and opsonic and haemolytic serum activities [35]. The innate immune mechanisms are specific for common structures in microbial groups but are unable to distinguish between small differences in exogenous pattern recognition receptors (PRRs), which include Toll-like receptors (TLRs), retinoic acid-inducible gene I-like receptors, nucleotide-binding oligomerization domain-like receptors and C-type lectin receptors [36]. These receptors recognize the pathogen-associated molecular patterns conserved in pathogenic organisms such as polysaccharides, lipopolysaccharides (LPS), bacterial DNA or viral RNA. These PRRs also recognize endogenous molecules released from damaged cells, known as damage-associated molecular patterns [36].

The adaptive immune response includes lymphocytes (B and T cells) as cellular components and secreted antibodies (immunoglobulins, Ig) as the humoral component [35].

Fish B cells are mainly produced in the head kidney, followed by the thymus and spleen [37,38,39,40]. Teleost B cells produce Ig to specifically label altered-host or foreign cells in order to agglutinate or precipitate soluble antigens, promoting phagocytosis [41]. In cartilaginous fish, three heavy chain isotypes have been detected: IgM, IgW, which has been referenced with many names (IgX, IgNARC or IgR) before it was proposed as an orthologue of IgD, and the lineage-specific isotype IgNAR [42,43,44,45,46]. Regarding teleost fish, three different Ig have been identified to date: IgM [47], IgD [48] and IgT/Z [49]. A tetramer of the IgM class containing eight antigenic combining sites is the most predominant Ig in teleost fish [47]. Based on the discovery of these three Ig in fish and their expression on B cells, several B cell subsets have been identified in different fish species pointing to a great diversity between them [39,50,51]. Moreover, the alternative splicing of pre-mRNA performed in fish (for review see [52,53]) suggests that the specific immune response of teleost fish might be as complex as in mammalian. Although there is no evidence concerning the presence of IgE, IgA or IgG in teleost fish, a functional FceRI receptor has been described [54], which, in mammals, acts as an IgE receptor. In mammals, T cells are categorized into two general populations: T cytotoxic (Tc) and T helper (Th) cells. The existence of Tc cells in fish, where they would be involved in specific cell-mediated cytotoxicity, has been suggested [55]. In fish, many molecules representing different Th cell subsets and their transcription factors have been demonstrated both at genetic and functional level [56,57]; however, further characterization of fish Th responses and the polarization of Th cells into Th subsets is needed [58,59]. As in mammals, the adaptive immune response of teleost T cells is thought to be mediated by the production of cytokines [41,60].

The aim of this manuscript is to review our present knowledge concerning the effect of sex steroids on fish leukocytes, taking into consideration that the studies performed vary as regards the fish species, doses, exposure protocols and hormones used.

## 2. Influence of Oestrogens on Fish Immune Responses

Oestrogens are involved in the regulation of oogenesis, vitellogenesis, testicular development and some other aspects of reproduction; in addition, they play important regulatory roles in many other systems (for review [61]). The immunomodulatory actions of oestrogens have been well documented for the mammalian immune system. In fish, this information is more limited although it has been known for many years that oestrogens regulate the immune system of fish [62,63,64,65,66] and several leukocyte functions [63,67,68,69,70,71]. Numerous studies have produced a substantial body of data concerning the effect of E_2_ on the fish immune response based on experimental in vivo and in vitro assays in which the activities of different immune cell types at different stages of their reproductive cycles were analysed (Table 1). The results permit to conclude that E_2_ influence the immune response of fish.

The effect of E_2_ on the immune response of gilthead seabream, a hermaphrodite fish species, has been widely studied, focusing on their leukocyte activities. In this species, the main phagocytic cell type, the acidophilic granulocytes, is strongly influenced by serum E_2_ levels although they do not express nuclear oestrogen receptors (ESRs). An increase in serum E_2_ levels promotes the movement of this cell type from the head kidney (the major haematopoietic organ in fish), as occurred after an inflammation response [72]. E_2_ also increases the transcription of some leukocyte adhesion molecules in vascular endothelial cells [65], promoting the recruitment of acidophilic granulocytes. Moreover, E_2_ enhance inflammation by increasing the production of a pro-inflammatory cytokine, the interleukin-1β (IL1β), in head kidney phagocytes [67]. In contrast to these data, head kidney macrophages of goldfish (*Carassius auratus*), previously treated with E_2_, show a decrease in their chemotactic response against endotoxin activated goldfish serum [69]. In gilthead seabream, E_2_ also has an inhibitory effect on acidophilic granulocytes as it inhibits their ROIs production activity [67]. Gilthead seabream macrophages also respond to E_2_, which alters the expression pattern of genes related with immunity [63]. As occurs in gilthead seabream, the in vivo E_2_ treatment inhibits the production of ROIs in rainbow trout (*Oncorhynchus mykiss*) and common carp (*Cyprinus carpio*) phagocytes [70,73] and nitric oxide (NO) production and phagocytic capability in common carp phagocytes [70]. However, the in vitro exposure of common carp and goldfish head kidney macrophages to E_2_ has no effect on ROI and NO production and slightly decreases their phagocytic capability [69,71]. This difference between the in vivo and in vitro effects in the same species might be explained by the data obtained for gilthead seabream that point to interactions between different immune cell types upon E_2_ treatment. Thus, macrophage conditioned media, obtained from E_2_ treated-macrophages, modify the phagocytic capability and ROIs production activity of head kidney cells in a similar way as E_2_ treatment [66]. Interestingly, the effect of E_2_ might be species-specific, as the effect is not the same in other fish species. For example, in Japanese sea bass (*Lateolabrax japonicus*), E_2_ increases the production of ROIs by blood macrophages and liver cells [74,75]. As regards pro-inflammatory cytokines, exposure of European sea bass juveniles to E_2_ for 35 days decreased the il1β and tumour necrosis factor *α* (tnfα) transcriptional levels and the IL1β content in serum. However, these effects were not constant with time [76].

Interestingly, chronic exposure to E_2_ during embryonic development, hatching and early larval development of rainbow trout fry leads to an impairment of the complement activation pathway upon a bacterial challenge and a concomitant decrease in the survival rate of the population exposed to E_2_ compared to non-exposed groups [77].

When the adaptive immune response is studied, species-dependent effects were also observed. Regarding total levels of IgM, they fell upon E_2_ treatment in gilthead seabream and rainbow trout (*Oncorhynchus mykiss*) but increased in Japanese sea bass [75,78,79,80]. Interestingly, in rainbow trout the amount of IgM-secreting cells was suppressed by E_2_ but were not affected in common carp [73,81]. Moreover, E_2_ impaired the mitogen-proliferation of peripheral blood leukocytes of goldfish in vivo and suppressed this activity in vitro in goldfish and catfish peripheral blood leukocytes [82,83].

Briefly, as it is not the main subject of this revision, 17α-ethynylestradiol (EE_2_), a pharmacological compound with strong estrogenic activity used in oral contraceptives and in hormone replacement therapy, is widely distributed in superficial waters, where it acts as an EDC in fish. The presence of EE_2_ in the European sewage and surface waters (ranging from 0.5 to 62 ng/L [84,85]) and the evidence of its effects in aquatic organisms, even at very low doses [86], have prompted to the European Union to include this substance in the monitoring programs of water pollution (document COM(2011)876; http://eur-lex.europa.eu/legal-content/EN/TXT/?uri=COM:2011:0876:FIN). Several in vivo and in vitro assays have amply demonstrated that EE_2_ might alter the capacity of fish to appropriately respond to infection although it does not behave as an immunosuppressor in juveniles or adult gilthead seabream [64,87,88]. In fact, EE_2_ triggered an inflammatory response in the peritoneal exudates of gilthead seabream [89]. Interestingly, the innate immune response of fish recover from the disruptive effects of EE_2_ [87,88].

### Oestrogen Receptor in Fish Leukocytes

Three nuclear ESRs (ESR1, ESR2a and ESR2b) have been cloned in most fish species studied (including gilthead seabream, Atlantic croaker, zebrafish, goldfish and European eel), while four ESRs (two ESR1 and two ESR2b) have been described in rainbow trout and *Spinibarbus denticulatus* [62,95]. A tissue-specific pattern of expression in fish immune tissues has been observed for nuclear ESRs (Table 2). In channel catfish, ESR1 is expressed in spleen, blood and head kidney, while ESR2 is only expressed in spleen [96]. ESR2 is expressed in the spleen and head kidney of common sole (*Solea solea*) [97]. In gilthead seabream, macrophages, lymphocytes and total peritoneal leukocytes express ESR1 while acidophilic granulocytes, as already mentioned, did not express any ESRs [66,89]. In European sea bass all ESRs have been identify in thymocytes at transcriptional levels and in thymocytes and mast cells at protein levels [98]. Interestingly, common carp head kidney monocytes/macrophages, neutrophils and lymphocytes expressed ESR1 and ESR2 but they were not found in naïve blood circulating leukocytes [99], suggesting that the effect of E_2_ on several immune cells also depend on the activation stage of leukocytes. The impairment of the ESR2b in a zebrafish mutant enhanced the susceptibility to anti-viral infections, although several genes related to the interferon pathway, including a negative regulator, were up-regulated in mutant fish [100]. However, these data do not explain the high loss in resistance upon viral infection recorded in mutant and compared with wild type fish, suggesting that anti-viral responses other than the interferon pathway might be affected by E_2_ signalling impairment.

It has been recognized for over 40 years that oestrogens, in addition to their classic genomic actions, can modulate several different signalling cascades in a non-genomic way [101]. Therefore, other receptors must also be involved because E_2_ actions have been described in cells lacking ESRs [102,103,104]. Despite the rapid effects of oestrogens had been identified earlier, it was not until 2005, that an orphan G-protein coupled receptor (GPCR) was identified as an oestrogen-binding intracellular membrane receptor [105,106,107].

In fish, European eel show two G-protein coupled oestrogen receptors (GPER: GPERa and GPERb) in reproductive tissues [108]. Although two GPERs are expected in most fish species as a consequence of genome duplication events that have occurred in fish, only one GPER has been characterized in most of the species studied, such as goldfish, gilthead seabream, Atlantic croaker, zebrafish, common carp and orange-spotted grouper (*Epinephelus coioides*) [7,99,109,110,111,112]. In gilthead seabream, GPER is expressed in acidophilic granulocytes but also in other leukocytes such as spleen and peritoneal leukocytes [89,109], although the correct identification of these leukocytes needs further studies. In acidophilic granulocytes, G1 (a specific agonist of GPER) promotes, in a very short time (less than 16 h), an anti-inflammatory effect both in vitro and in vivo mainly in naïve cells and non-vaccinated fish. Interestingly, GPER hardly modifies the ROIs production of acidophilic granulocytes [109]. In addition, GPER signalling in vivo modulated the adaptive immunity in gilthead seabream [109]. E_2_ modulate vertebrate granulocyte function through a GPER via PI3K in common carp macrophages [99] or through cAMP/protein kinase A/CREB signalling pathways in gilthead seabream granulocytes [109].

## 3. Influence of Androgens on Fish Immune Responses

The effect of androgens on the immune system of fish has been less studied than the effect of oestrogens (Table 1). The androgen system of fish is quite complex, due to the fact that T is the main metabolite to be transformed into 11KT, OHT or DHT [14]. All of them have androgenic effects on fish reproductive tissues and have a role in the regulation of gametogenesis [118]. Moreover, DHT can also be transformed into 5α-androstane-3β, 17β-diol (β-diol), which has estrogenic activities [119]. In fish, these transformations occurred in several tissues including the testes, brain and liver [14,118]. The in vivo administration of T modifies the serum levels of other androgens and even those of E_2_. Thus, T administration increases T and 11KT and decreases DHT levels in serum of gilthead seabream [120]. As androgens are transformed in several tissues, the effect observed upon the exogenous administration of one of them could be due to the administered androgen or to the increase or decrease in the amount of any of the others into which the administered androgen could be transformed. For that, the importance of each androgen in the regulation of the immune response using in vivo experiments is not easy, as might be expected. In that sense, some in vitro data might help to clarify this issue. As in the case of oestrogens, many aquatic pollutants disrupt androgen signalling in fish [6].

### 3.1. Testosterone

The immunocompetence-handicap hypothesis is that T inhibits the immune response in order to guarantee the health of those specimens with well-developed testosterone-dependent sexual signals and that the activation of an immune response leads to a decrease of T serum levels [121]. In salmonids, the immunosuppression observed in some stages of the reproductive cycle has been linked to androgens since leukocytes display a specific androgen receptor (AR) [114]. In fact, in vitro exposure of salmonid leukocytes to T decreases the ability of head kidney lymphocytes to form specific antibody producing cells [94] and triggered the death of total head kidney leukocytes [122]. In rainbow trout, T also causes a reduction in IgM secreting cells in peripheral blood, head kidney, spleen and skin leukocytes [79].

However, as data about the effect of T on different fish species and immune tissues are accumulated, the empirical evidence supporting the immunocompetence-handicap hypothesis in fish becomes weaker. In tench (*Tinca tinca* L.) the activation of an immune response upon β-glucan exposure decreased the serum levels of T that was predicted by the immunocompetence-handicap hypothesis [92]. However, T did not suppress the lysozyme activity of plasma or the production of ROIs by blood leukocytes and head kidney phagocytes [92] as also occurs with the phagocytic activity of peripheral blood of tilapia and common carp when treated in vitro with T [68]. In fact, in common carp, T has tissue-dependent effects on leukocytes as it sharply reduced the number of IgM secreting cells and IgM production in splenic leukocytes but not in circulating blood and head kidney leukocytes [81].

Surprisingly, the innate immune system of gilthead seabream is stimulated by T. Thus, in vivo an increase of serum T levels triggered high complement and peroxidase activity levels [78]. T also primed the phagocytosis and ROIs production activities of head kidney leukocytes in vitro [91] and in vivo [90]. In fact, the transcription levels of interleukin 1β, *il1b* and some *tlrs* genes are up-regulated in T-exposed head kidney leukocytes in vivo [90], as also occurred when professional phagocytic cells were isolated and treated with T in vitro [91].

### 3.2. 11-Ketotestosterone

As 11KT is the main androgen in fish, most studies into androgen regulation of the immune response have used this androgen. In rainbow trout, 11KT triggers the same effect as T, decreasing the number and capability of IgM-secreting cells of spleen, head kidney, blood and skin [79]. ROIs production and the phagocytic activity of common carp head kidney macrophages also decreased by 11KT [70]. However, when peripheral blood phagocytic activity after 11KT exposure was analysed, no effects were observed in common carp or tilapia [68], nor in the apoptotic rate of splenic or peripheral blood leukocytes of common carp [123]. In gilthead seabream, 11KT is able to increase proIL-1β accumulation and the ROIs production activity of non-stimulated head kidney phagocytes [67]. However, 11KT impairs the activation of the ROIs production by total head kidney leukocytes upon challenge with bacterial DNA in vitro [91]. Moreover, acidophilic granulocytes and macrophages of gilthead seabream did not respond equally to 11KT. Thus, 11KT was quite effective at decreasing the gene expression of several *tlrs* in isolated acidophilic granulocytes activated or not with bacterial DNA, while in activated or non-activated macrophages the expression of *il1b* and *tlrs* increased in a dose-dependent manner at most of the doses used [91]. Interestingly, when macrophages are treated with T and 11KT simultaneously, T inhibits the up-regulation of *il1b* and *tlr9* genes induced by 11KT [91]. In male three-spined sticklebacks (*Gasterosteus aculeatus*), a negative correlation between 11KT serum levels and ROIs production in the phagocytosis assay was observed [94].

### 3.3. Other C-19 Steroid with Androgenic Function

In fish, DHT and OHT are also produced from T and in some species, they are the main androgens found in plasma or testis. Thus, in urohaze goby (*Glossogobius olivaceus*), the main androgen is DHT, which is produced by T conversion in several tissues including brain and gonad. Recent studies have demonstrated that DHT has androgenic functions in juvenile fathead minnows (*Pimephales promelas*) where it triggers testicular development through the first spermatogenetic wave and the appearance of intersex in females [124]. Moreover, in male gilthead seabream specimens, the increase in DHT triggers the meiotic phases of spermatogenesis, which seems to be regulated by E_2_ and T serum levels [120]. The OHT is also a potent androgen in some fish species, which is produced in gonad, liver and even blood cells [125,126]. There are not studies that relate DHT or OHT with the immune system. However, taking into account fresh data about the ability of these androgens to activate ARs [127], such studies would be performed and will probably shed more light on the complex and controversial data related with androgen effect on leukocytes.

### 3.4. Androgen Receptors in Fish Leukocytes

In salmonids the presence of ARs has been described in total head kidney leukocytes of rainbow trout [114]; however, little further information has been accumulated. Two nuclear ARs (ARa and ARb) have been described in cDNA libraries or in transcriptional studies performed in the testes of several fish species [128]. ARs expression has been reported in immune competent organs (Table 2), such as the head kidney, liver and spleen of sea bass [115] and zebrafish [116]. Recent studies performed in gilthead seabream described the presence of an AR in macrophages and acidophilic granulocytes [91]. It should be noted that all the in vitro data obtained in gilthead seabream macrophages and acidophilic granulocytes were determined only upon 3 h of stimulation with androgens, suggesting the existence of a membrane androgen receptor (mAR), as occurs in mammals [129]. However, in fish this membrane receptor has not been described to date. Mammalian AR is transcriptionally modified to form several variants of the ARs that have differential expressions and functions, mainly in cancer cell lines [129]. In gilthead seabream a splice-variant of the AR, the ARΔLBD variant, occurs in acidophilic granulocytes but not in macrophages [117], while AR is expressed in both cell types [91]. T up-regulate the AR and ARALBD transcription in acidophilic granulocytes [91,117]; the ARΔLBD/AR ratio is positively correlated with T serum levels [117]. Furthermore, the activation of acidophilic granulocytes with bacterial DNA modulates the ARΔLBD/AR ratio in a reproductive stage-dependent way [117], inducing a decrease during spermatogenesis stage and an increase during spawning. Although further studies are needed, the existence of several variants of ARs in fish leukocytes, together with the affinity of all the androgens previously described to ARs, may lead to an array of different sensitivities in the cells to different androgen levels.

## 4. Influence of Progestins on Fish Immune Responses

Natural progestins regulate several reproductive processes in vertebrates. A major progestin in fish teleost is 17α,20β-dihydroxy-4-pregnen-3-one (DHP), which has been seen to be involved in sperm hydration and the activation of motility in some fish species [15,16]. Another related progestin is 17α,20α-dihydroxy-4-pregnen-3-one, the spermation-inducing hormone in amphibian species [130]. However, progestins also have an essential role in early gametogenesis, triggering the meiosis of male germ cells [17]. Synthetic progestins are used in humans as part of contraceptive therapies and it has also been claimed that they mainly impair T cell functions. These progestins inhibit the production of T-cell derived factors and alter the subset populations of T cells and their ratios. Moreover, the progestin, medroxyprogesterone (MPA), blocks E_2_ pro-inflammatory effects in several tissues such as injured vessels, endometrium and cervix [131,132]. In fish, however, to the best of our knowledge, two studies pointed to a role for progestins in the immune response of fish. In carp (*Cyprinus carpio*), DHP and MPA inhibit NO release by activated leukocytes as well as down-regulate the transcription of pro-inflammatory type I immune related-factors [133]. Interestingly, in tilapia (*Oreochromis niloticus X O. aureus*) progesterone had no suppressive effect on phagocytosis activity [68].

### Progestins Receptors in Fish Leukocytes

Nuclear and membrane progestin receptors have been characterized in the reproductive tissues of several fish species. For example, 5 membrane receptors and 2 nuclear receptors have been described in European eel [108]. Interestingly, in reproductive tissues and brain, two different membrane proteins have been described [134]. The first one is the progesterone membrane receptor (mPR) that displays high affinity and specificity for progestin binding and promotes progestin signalling in vertebrate tissues, including fish. The second one is the progesterone receptor membrane component 1 (PGMRC1), which belongs to the membrane-associated progesterone receptor family and has been described in mammals [134]. This evidence points to a very complex network of receptors that leads to progestins biological activity in reproductive tissues. As fish leukocytes display a wide array of different hormone receptors [135,136], the presence of progestin receptors seems to be feasible and further studies on progestin and its receptors on fish leukocytes need to be performed in order to have a better understanding of the effect of sex steroids on the immune system of fish.

## 5. Immune System in the Fish Gonad

The gonad is the main steroidogenic organ in vertebrates and the levels of sex steroids in this organ may be even higher than those found in serum [137]. The gonad is considered an immune-privileged organ due to the ability of foreign tissue allografts to survive inside this organ, as the immune response is not activated against them. However, although physical barriers are present in the testis of all vertebrates, this is not the main mechanism underlying this phenomenon. This special status is mainly due to active local mechanisms of regulation that suppress the activation of leukocytes [138]. In fact, acidophilic granulocytes of gilthead seabream come into close contact with germ cells at certain stages of the reproductive cycle without triggering any inflammatory response and without phagocytizing them [139]. Moreover, leukocytes located in mammalian gonads orchestrate important reproductive physiology processes, including gametogenesis and steroidogenesis [140,141]. Much time has passed since leukocytes were first described in the gonad of teleosts. Since them, several types of leukocytes have been described in the testis of different teleost species using light and electron microscopy, while functional analysis has also been applied to gonadal leukocytes in several fish species. Thus, changes in the number and localization of leukocytes have been described in several fish species related to stages in the reproductive cycle [142]. The leukocytes described are macrophages, granulocytes, histamine positive cells and lymphocytes [67,143,144,145]. In fact, both IgM and IgT gene expression has been observed in fish testes [146], suggesting the presence of two different subsets of B lymphocytes. Interestingly, the acidophilic granulocytes are actively recruited by the gonad in physiological and non-pathological conditions through the regulation of adhesion molecule transcription and the production of chemiotactic factor [22,139]. In fact, when oestrogen or androgen serum levels are experimentally increased, the rate of leukocyte recruitment in the gonad is modified [137,146]. In rainbow trout a fish specific chemokine receptor, which has no equivalent in humans, is transcripted in the gonad [147]. Interestingly, the epigonal organ is a lymphomyeloid organ present in the elasmobranch gonad, whose haematopoietic activity seems to be correlated to hormone levels [148]. Moreover, the amount of leukocytes (macrophages, neutrophils and lymphocytes) presents in the ovarian cavity of *Neoditrema ransonneti*, a viviparous teleost species, are related with the reproductive cycle stages independently of the presence of semen in this cavity [149]. Functional data reveal that gonadal leukocytes of gilthead seabream have a specific pattern of activation completely different to the pattern shown by their head kidney counterparts. Thus, testicular acidophilic granulocytes have very low levels of ROIs production and a heavily suppressed phagocytic activity, while they constitutively express and accumulate IL1β [67,139]. The metalloprotease profile displayed by testicular acidophilic granulocytes is also different from their head kidney counterparts [150]. All these data suggest that fish leukocytes might be very sensitive to sex steroid levels and are crucial to our understanding of this process so that their sensitivity to pollution by EDCs can be properly assessed.

The special regulation of the immune response in gonad is particularly relevant in the analysis of pathogens that may be transmitted to the following generation through the ovarian and seminal fluids or even inside the gametes (vertical transmission). Interestingly, several infectious pancreatic necrosis virus (IPNV)-detecting assays have reported differences when the samples were processed at different stages of the reproductive cycle [151], strongly suggesting an interaction between reproductive parameters and the virus life cycle and probably interference in the immune regulation of the gonad. Thus, several studies have determined and characterized the gonadal immune response upon virus colonization using different fish/virus models. IPNV and viral haemorrhagic septicaemia virus (VHSV) colonized the ovary of rainbow trout, although only VHSV actively produces enough mRNA and protein to be detected, while IPNV triggered a latent infection of the ovary [152]. In turn, VHSV strongly induces the activation of the transcription rates of several chemokines, interferon and myxovirus (influenza) resistance protein (mx) genes, while IPNV neither elicits nor inhibits these responses [152]. Interestingly, the suppression of the immune response upon IPNV colonization of the ovary seems to be related to the exogenous systemic factors produced during IPNV infection [153]. A different immune response has been reported in the testis of European sea bass and gilthead seabream specimens upon NNV infection [154]. In general, the immune response was induced in European sea bass, while in gilthead seabream a viral mRNA and protein production was detected in testis [154]. The rapid immune response elicited in the gilthead seabream brain (target tissue of NNV) and not in the gonad, allows the specimens to survive the viral infection and to become carrier of the virus. Interestingly, the production of E_2_ and 11KT was altered upon NNV infection [154], suggesting that the virus might alter the reproductive function in order to improve its own transmission or the immune response as sex steroids also regulated inflammation and other immune responses (see above). In fact, a positive correlation between the transcription levels of ESRb2 and several ARN sensor, intermediate and effector molecules of the interferon (IFN) pathway in the testis of European sea bass [155], the implications of which are being studied in our laboratory.

## 6. The Effect of Other Hormones on the Immune Function

Although this is not the main scope of this review, we mention many other biological processes in which a clear connection between hormones and immune functions exists. Moreover, leukocytes display multiple hormone receptors that modulate their activation and functions and, in turn, the immune response [135].

The response to stress involves a range of mechanisms that allow to readjust the homeostasis of an organism that has been altered upon the action of an intrinsic or extrinsic stressors [156]. The release of cortisol into blood stream is one of the main indicators of stress in fish [156]. Cortisol is involved in the regulation of the immune response but also regulates reproduction. The regulatory pathways of sex steroids and cortisol production are known to be interconnected [157]. Thus, cortisol induces immunosuppression but also regulates sex reversal by means of which environmental conditions can altered the fish sex determination and induce the development of another sexual phenotype [157]. In addition, it has recently been described that the sensitivity of leukocytes to sex steroids is regulated by cortisol and stress conditions through regulation of the transcription and production of ESRs, GPER1 and local aromatase [158].

Another interesting biological process related with reproduction is the smoltification of salmonids, by means of which, the specimens are able to migrate from freshwater to marine water, while adapting their biological processes to salinity [159]. This process is related with puberty and is orchestrated by hormones such as growth hormone, cortisol and thyroid hormones [135]. Interestingly, although increases in 11KT, T and E_2_ serum levels have been observed during smoltification in salmonids, the exogenous administration of these hormones inhibited spring time smoltification in masu salmon [160]. Immunosuppression has been observed during smoltification, which is related with high levels of cortisol [159]; however, further studies are needed to expand our knowledge about this developmental process, which is critical for the improvement of salmonids aquaculture by preventing diseases and pathological losses.

## 7. Conclusions

In conclusion, the data accumulated to date reveal that fish leukocytes are sensitive to sex steroids. As most of the studies have been carried out with oestrogens or analyzed oestrogen regulation, to date is possible to conclude that they modulate the fish immune response. However, although some data about the influence of androgens and progestins on the fish immune response exists, more studies are needed to have a clear overview of this process. Taking into account that the immune response is essential for fish survival mainly during critical biological process such as stress responses, smoltification, sex change and reversal or even the blockage of viral transmission through the gonad, a better understanding of this regulation will improve fish production.

## Figures and Tables

**Table 1:** Effects of 17β-estradiol (E_2_), testosterone (T) or 11-ketotestosterone (11KT) on different types of leukocytes using in vivo and in vitro experiments in different teleost species.

Sex Steroids	Leukocytes	Treatment	Effects	Fish Species	References
E_2_	Head kidney cells	In vivo	Decrease of IL1β and TNFα transcription and IL1β production	European sea bass	[76]
		In vitro	Increase of IL1β production	Gilthead seabream	[67]
		In vitro	Inhibition of ROIs production activity	Gilthead seabream	[67]
	Head kidney acidophilic granulocytes	In vivo	Migration from head kidney to peripheral tissues	Gilthead seabream	[72]
	Macrophages	In vivo	Inhibition of ROIs production activity	Rainbow trout	[73]
	Blood macrophages	In vivo	Increases of ROIs production activity	Japanese sea bass	[75]
	Phagocytes	In vivo	Inhibition of NO production	Common carp	[70]
		In vivo	Inhibition of ROIs production activity	Common carp	[70]
	Head kidney macrophages	In vitro	Inhibition of chemotaxis against endotoxin	Goldfish	[69]
		In vitro	Impartment of the immune-related gene expression pattern	Gilthead seabream	[63]
		In vitro	Non-effect on ROIs and NO production	Common carp	[71]
		In vitro	Non-effect on ROIs and NO production	Goldfish	[69]
		In vitro	Inhibition of the phagocytic capability	Common carp	[71]
		In vitro	Inhibition of the phagocytic capability	Goldfish	[69]
	Peripheral blood leukocytes	In vitro	Suppression of mitogenic activity	Goldfish	[83]
		In vitro	Suppression of mitogenic activity	Channel catfish	[82]
		In vivo	Impairment of mitogenic activity	Goldfish	[83]
	IgM-secreting cells	In vivo	Impairment of mitogenic activity	Goldfish	[83]
		In vivo	Decreases on IgM production	Gilthead seabream	[78]
		In vivo	Decreases on IgM production	Rainbow trout	[73]
		In vivo	Increases on IgM production	Japanese sea bass	[75]
	Head kidney leukocytes	In vivo	Increases of IL1β and TLRs transcription	Gilthead seabream	[90]
		In vitro and in vivo	Increases on ROIs production	Gilthead seabream	[90,91]
		In vitro	Non-effect on ROIs production	Tilapia	[68]
		In vitro	Increases on phagocytosis	Common carp	[68]
		In vitro		Gilthead seabream	[91]
	Acidophilic granulocytes	In vitro	Increases of IL1β and TLRs transcription	Gilthead seabream	[91]
	Blood leukocytes	In vivo	Non-effect on lysozyme activity	Tench	[92]
		In vivo	Non-effect on ROIs production	Tench	[92]
	IgM-secreting cells	In vitro	Decreases in number in blood, head-kidney, spleen and skin	Chinook salmon	[93]
		In vitro		Rainbow trout	[80]
		In vitro	Reduction in spleen	Common carp	[81]
			Non-effect in head-kidney		
			Non-effect in blood		
11KT	Head-kidney macrophages	In vivo	Inhibition of ROIs production	Common carp	[70]
		In vivo	Inhibition of phagocytosis	Common carp	[70]
		In vitro	Increases of TLRs and IL1β transcription	Gilthead seabream	[91]
	Blood leukocytes	In vitro	Non-effect on phagocytosis	Common carp	[68]
		In vitro		Tilapia	[68]
	Head kidney phagocytes	In vitro	Activation of ROIs production	Gilthead seabream	[67]
		In vitro	Increases pro-IL1β accumulation	Gilthead seabream	[67]
		In vivo	Inhibition of ROIs production	Three-spine sticklebacks	[94]
	Head kidney acidophilic granulocytes	In vitro	Decreases of TLRs transcription	Gilthead seabream	[91]
	IgM-secreting cells	In vivo and in vitro	Decreases production	Rainbow trout	[79,80]
		In vitro	Decreases in number in blood, head-kidney, spleen and skin	Rainbow trout	[80]

**Table 2:** Presence of different sex steroid receptors on leukocytes and immune tissues of different teleost species.

Sex Steroid	Receptor	Tissue or Cells	Fish Specie	References
E_2_	ESR1	Spleen, blood and head-kidney cells	Channel catfish	[113]
		Macrophages	Gilthead seabream	[66]
			Common carp	[99]
		Neutrophils	Common carp	[99]
		Lymphocytes	Gilthead seabream	[66]
			Common carp	[99]
		Thymocytes	European sea bass	[98]
		Mast cells	European sea bass	[98]
		Peritoneal leukocytes	Gilthead seabream	[89]
	ESR2	Spleen	Channel catfish	[113]
		Spleen and head-kidney	Common sole	[97]
		Macrophages	Common carp	[99]
		Neutrophils	Common carp	[99]
		Lymphocytes	Common carp	[99]
		Thymocytes	European sea bass	[98]
		Mast cells	European sea bass	[98]
	GPER	Macrophages	Common carp	[99]
		Acidophilic granulocytes	Gilthead seabream	[109]
		Peritoneal leukocytes	Gilthead seabream	[89]
Androgens	AR	Head-kidney	salmonids	[114]
			European sea bass	[115]
			Zebrafish	[116]
		Liver	European sea bass	[115]
			Zebrafish	[116]
		Spleen	European sea bass	[115]
			Zebrafish	[116]
		Macrophages	Gilthead seabream	[91]
		Acidophilic granulocytes	Gilthead seabream	[91]
	ARΔLBD variant	Acidophilic granulocytes	Gilthead seabream	[117]
